# On the Role of Psychoneuroimmunology in Oral Medicine

**DOI:** 10.1016/j.identj.2022.07.002

**Published:** 2022-09-30

**Authors:** Lennart Seizer, Christian Schubert

**Affiliations:** a Department of Psychiatry, Psychotherapy, Psychosomatics and Medical Psychology, Medical University Innsbruck, Innsbruck, Austria; b Institute of Psychology, University of Innsbruck, Innsbruck, Austria

**Keywords:** Psychoneuroimmunology, Psychoneuroendocrinology, Periodontitis, Oral lichen planus, Herpes labialis, Integrative single-case study

## Abstract

Psychoneuroimmunology (PNI) is an area of interdisciplinary research exploring the complex interactions within the immuno-neuro-endocrine system in response to psychosocial influences. Such influences can trigger neurological changes, leading to immunological effects related to the emergence and course of various diseases. This concise clinical review explores the role of PNI in oral medicine in three exemplary models of oral disease: periodontitis, herpes labialis, and oral lichen planus. Previous literature has shown that psychosocial stress is related to exacerbations in these three oral diseases and to poorer overall oral health. The presumed biological mechanisms affect the activity of stress axes, i.e. the hypothalamus-pituitary-adrenal (HPA) axis and the sympathetic nervous system (SNS), and subsequent immune system dysregulation. Although these PNI mechanisms remain poorly understood, several stress reduction interventions in clinical oral medicine have already yielded promising results. In future work, the elucidation of pathways within PNI networks will require carefully designed studies with sensitive methodology, e.g. the integrative single-case design. A biopsychosocial approach has the potential to move disease models in oral medicine from simple connections rooted in empirical dualism and reductionism to the establishment of network-based models. Further research on these complex connections should lead to novel clinical approaches and preventive strategies in oral medicine.

## Introduction

### Systems medicine

A modern, comprehensive approach to medicine requires a systemic perspective on humans. It integrates and analyses multimodal data from biological, psychological, and social entities in their dynamic response to adaptive stimuli. The network models generated in this process can provide insight into underlying divergences between health and disease.^1^ In so doing, the organism can be understood as a functional system of organs, tissues, cells, and molecules inextricably embedded in higher-level psychosocial and cultural entities and constantly influenced by them.^2^^,^^3^ A biopsychosocial systems approach could move disease models in medicine from simple connections rooted in empirical dualism and reductionism to the establishment of network-based models in which cybernetic processes and emergent phenomena are adequately accounted for.^1^^,^^4^

**Fig. 1:**
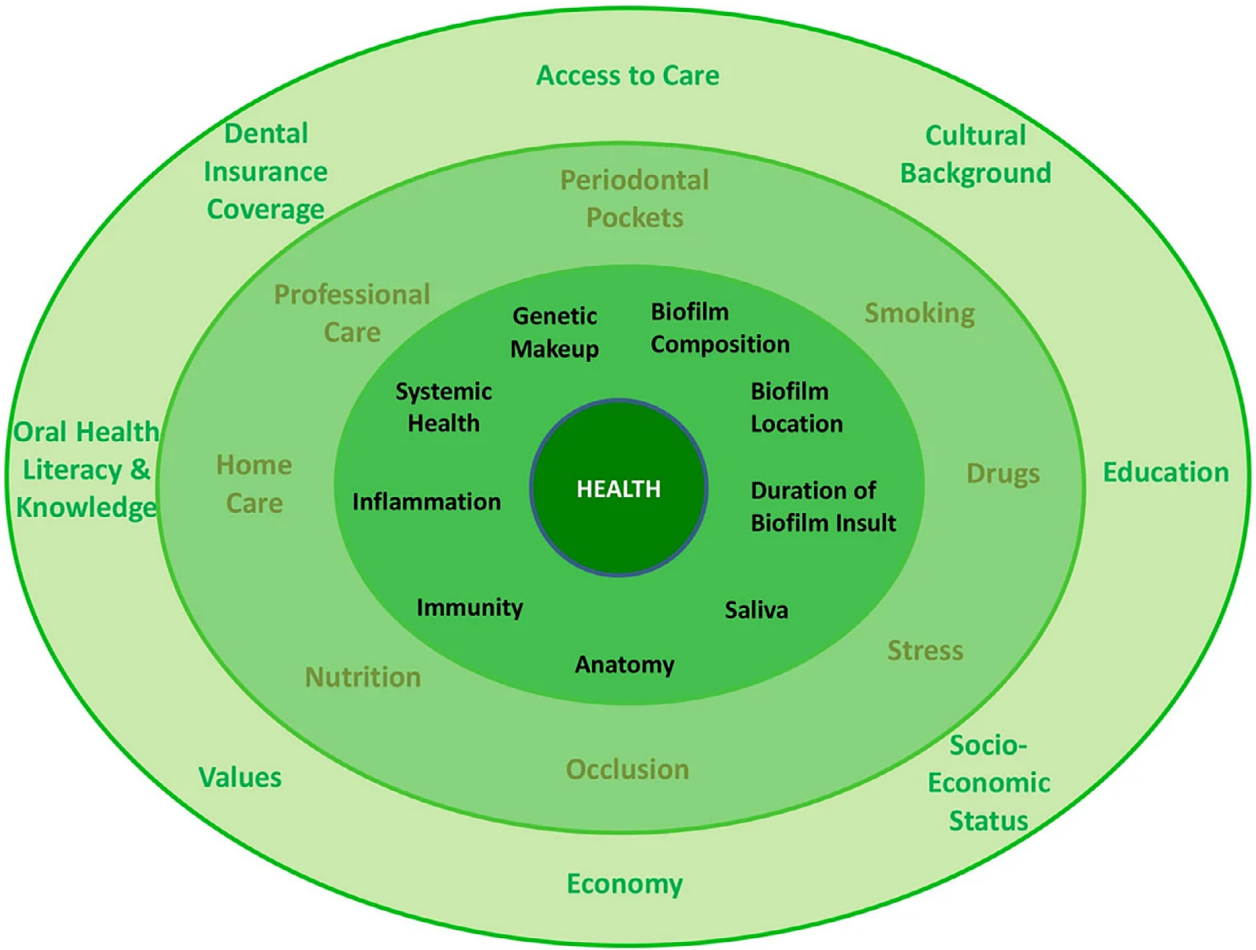
The 3-layered periodontal health model (with the kind permission of Mariotti and Hefti^5^).

Mariotti and Hefti^5^ have transferred some of these ideas into a general model on the determinants of periodontal health and disease (Figure 1). In this model, influencing factors are divided into 3 different layers: first, biological entities with a direct impact such as oral biofilm composition and characteristics, immune system activity within the oral cavity, genetic predisposition, and overall systemic health and disease; second, environmental and systemic factors that can influence biological components (eg, nutrition, drug use, stress, oral hygiene); and third, general conditions of an individual that affect both biological and psychosocial factors, such as cultural and biographical background, socioeconomic status (SES), and access to professional care.

The factors presented in this model are highly intertwined in terms of their occurrence, intensity, and effect. The influence psychological stress exerts on oral health, for example, is first dependent on the cognitive-affective appraisal given to specific potentially stressful incidents, which in turn can be affected by an individual's cultural background, education, and SES. Second, during states of stress, individuals are prone to coping behaviour potentially associated with negative impacts on oral health, such as smoking, poor nutrition and inadequate home care. Third, the physiological stress response and its effect on oral health, both of which will be discussed in more detail in the following section, is dependent on the condition of the organism at the time of occurrence – for example, on systemic health and immune system activity.^5^

### Psychoneuroimmunology

Psychoneuroimmunology (PNI) can be understood as the empirical realisation of the biopsychosocial paradigm in medicine.^4^ In this context, PNI, as an interdisciplinary field of research, investigates the complex interactions amongst psychological, neuronal, endocrine, and immunologic processes.^6^ Milestones in this field are findings on the multiple interaction and communication pathways between different physiological systems. For example, it has been shown that psychological, neuronal, and hormonal activities influence the immune system.^7^, ^8^, ^9^ In the opposite direction, cytokines can act on endocrine glands and neurons and alter an individual's subjective experience and behaviour.^9^^,^^10^ Other milestones in PNI concern the biochemical basis for reciprocal connections between the psyche and the immune system, such as the “immuno-neuro-endocrine network”^11^ and the “common biochemical language.”^12^

A central topic in PNI, both in research and the clinical field, is the psychophysiological processing of stressors. Stress refers to the mental and physical reactions caused by specific stimuli (stressors) to cope with particular demands in subjectively aversive situations.^13^ A significant role in the differentiation of stress is attributed to the perceived controllability and predictability of a stressor.^14^ The term *stress* is used in the following in the sense of distress, that is, to describe situations that exceed the resources of individuals in a maladaptive manner and are accompanied by negative emotions and associated physical reactions.

At the beginning of the stress reaction, potentially stressful events are evaluated in cognitive-affective processes. These assessments can differ considerably from person to person. Like other subjective phenomena, the experience of stress is very much tied to an individual's current and past experiences. Stress axes are activated after the subjective assessment of a stressor and its central nervous processing in different brain areas (eg, prefrontal cortex, hippocampus, amygdala) to prepare the organism for upcoming challenges. The two best-described stress axes are the sympathetic nervous system (SNS) or sympatho-adrenomedullary (SAM) axis, upon activation of which catecholamines (epinephrine, norepinephrine) are released, and the hypothalamic-pituitary-adrenal (HPA) axis, which responds to stress by releasing cortisol. Both stress axes are associated with several target organs and a wide range of stress-related peripheral effects.^15^^,^^16^

Concerning the immune system, acute stress is usually associated with an initial increase in inflammatory activity controlled by catecholamines. This is likely a protective mechanism to immunologically counteract possible injury or infection quickly.^16^ However, since a disproportionate or excessive inflammatory response would damage the body, the HPA axis and thus cortisol release is stimulated in the further course of the stress response. This occurs primarily via peripherally released pro-inflammatory cytokines, including interleukin (IL) 1, IL-6, and tumour necrosis factor-alpha (TNF-α). These cytokines form of a self-regulatory feedback loop in that they all act on multiple levels of the HPA axis e.g. the paraventricular nucleus (PVN; the starting point of the HPA axis), the pituitary and the adrenal gland.^16^, ^17^, ^18^

From an evolutionary standpoint, this stress response has contributed to an individual's survival in dangerous situations. However, this response is often inappropriate given the nature of stressors in modern lifestyles—especially psychosocial stress, such as occupational overload, financial worries, and relationship problems. Specifically, the stress system is activated over more extended periods, which can be accompanied by disturbances of various bodily functions.^16^ For example, due to chronic (intermittent) stress, dysregulation of the HPA axis may occur, characterised by sustained elevated cortisol levels, sometimes for years, that is, hypercortisolism (overreactive HPA axis; hypercortisolemia). In the immune system, hypercortisolism can lead to impaired production of cytokines; loss of lymphoid, thymic, and splenic tissue; and systemic suppression of cellular immunity. This, in turn, is associated with an increased risk of intracellular infection, wound healing disorders, and cancers.^19^, ^20^, ^21^, ^22^

Over time, maladaptative processes occur in order to deal with stress-induced hypercortisolism and the compensatory inhibition of the immunoregulatory cortisol effect. This can lead to permanently decreased cortisol levels, referred to as hypocortisolism (insufficiently responsive HPA axis; hypocortisolemia).^19^^,^^21^ In addition, glucocorticoid receptor (GR) activity, expression, and signal transduction may change in the setting of GR resistance, affecting the action of cortisol in target tissues and cortisol-induced HPA axis downregulation. Both hypocortisolism and GR resistance are, in turn, associated with increased inflammatory activity and corresponding tissue damage, which may lead to the development of stress-associated inflammatory diseases in the long term.^19^^,^^22^

## PNI in oral medicine

Multiple studies have already demonstrated links amongst psychosocial factors, the immune system, and oral health.^23^, ^24^, ^25^, ^26^ Specifically, psychosocial stress has been associated with poorer oral health. However, the psychophysiological pathways involved are still largely unclear. The relevance of direct immuno-neuro-endocrine effects was demonstrated via various biomarkers in the saliva of healthy patients (eg, α-amylase, TNF-α, IL-6, IL-1β). Such biomarker concentrations changed after specific psychological influences, indicating a direct biochemical effect of psychosocial events on the oral milieu.^27^ In addition, certain behaviours that occur primarily during stress, such as decreased dental hygiene, unhealthy diet, and smoking, may also harm oral health.^24^ These behavioural effects can be difficult to distinguish from direct nonbehavioural immuno-neuro-endocrine effects. Additionally, it can be difficult to establish causal links between stress and the occurrence of oral diseases because the latter are often accompanied by pain or visible lesions. These symptoms affect patients’ quality of life and may act as stressors themselves.^28^ The following provides an overview of the current state of research on psychosocial influences and immuno-neuro-endocrine interactions in 3 exemplary oral diseases: periodontitis, herpes labialis, and oral lichen planus (OLP).

### Periodontitis

Periodontitis, which belongs to the group of periodontal diseases, is a chronic inflammation of the tooth-supporting tissue (periodontium) and can be expressed in intermittent relapses and continuous progression. The main pathologic features are clinical attachment loss, alveolar bone loss pocket formation, and gingival inflammation.^29^ Interactions between infectious bacteria and a dysregulated immune response appear to be essential in the aetiology of periodontitis. However, it is unclear whether colonisation by specific bacterial strains or the inflammatory response occurs first. Accordingly, periodontitis cannot yet be classified as an infectious or as an inflammatory disease.^30^, ^31^, ^32^ A systematic review of research dealing with the influence of psychological factors on susceptibility to periodontal disease found a positive association in most of the studies analysed (57.1%); 28.5% of the studies showed an ambivalent association (ie, only some psychological variables were associated with periodontal disease), and 14.2% of the studies showed no association between psychological factors and periodontal disease.^25^ Psychological variables that were associated with the occurrence and progression of periodontal disease or periodontitis were psychosocial stress,^33^, ^34^, ^35^, ^36^ depression,^24^^,^^37^ anxiety,^36^, ^37^, ^38^ loneliness,^39^ maladaptive coping,^40^ and stressful life events.^41^^,^^42^

A possible psychoneuroimmunological link between psychosocial stress and periodontitis may lie in the increased activation of the HPA axis, which inhibits cellular immune activity via increased cortisol release.^18^ Wound healing in soft tissues, such as the periodontium, is impaired—that is, slower—in patients with increased levels of cortisol^43^ or epinephrine.^44^ This impaired wound healing can increase the risk of infection or further injury.^45^ Stress-induced immunosuppression may also favour bacterial infections, which, in turn, cause destructive periodontitis.^25^^,^^45^ The relationship amongst psychosocial stress, salivary cortisol concentrations, and the severity of periodontitis has already been demonstrated in humans.^33^^,^^46^ Moreover, an animal experiment in rat strains with different degrees of HPA axis responsiveness showed that increased HPA axis activity is associated with increased corticosterone and greater destruction of periodontal tissues. This suggests a positive feedback loop between HPA axis activity and periodontitis.^47^ However, more (longitudinal) studies using sensitive methodological approaches are necessary to further elucidate the relationship between periodontitis and the HPA axis and its effector cortisol.^48^ In addition, excessive or prolonged stress may be associated with more severe immune system dysregulation with regard to systemic inflammation,^19^^,^^24^^,^^49^ which is thought to be the link between periodontitis and common comorbidities of periodontitis, such as coronary artery disease, osteoporosis, diabetes, and immune disorders (eg, rheumatoid arthritis).^50^, ^51^, ^52^

### Herpes labialis

Herpes labialis is caused by infection with the herpes simplex virus type 1 (HSV-1) and is associated with blistering of the lips and perioral area (“cold sores”). In rare cases, the disease is associated with fever and additional constitutional symptoms such as headache, myalgia, and malaise. The virus remains latent in the trigeminal ganglion after primary infection and can be reactivated when cellular immune activity decreases.^53^ Many studies have shown that psychosocial stress, in addition to other factors (eg, sunlight, physical exertion), is a major risk factor for symptomatic HSV-1 recurrence. In this connection, stress hormones (eg, glucocorticoids, catecholamines) likely mediate between psychological stress and herpes recurrence as they can alter the activity of specific memory T cells, dendritic cells, and natural killer cells.^54^, ^55^, ^56^

Using a research approach with weekly measurements over 32 weeks, Schmidt et al found a decrease in CD4^+^ T cells in the initial phase of HSV-1 relapse^57^ and, in another study, an increase in daily stress, stressful life events, and anxiety 1 week before relapse.^58^ Inhibition of cellular immunity can lead to viral reactivation, which is compensated by an increase in specific antibodies against newly formed viral protein.^59^ This is reflected in findings showing that adolescents who experienced physical violence at a young age or grew up in an orphanage during the first years of life had significantly elevated levels of HSV-1–specific antibodies in saliva compared to adolescents who came from more favourable family environments.^60^ Furthermore, it has been shown that the ability to neutralise HSV-1 in the saliva of women who experienced physical and psychological abuse in their intimate relationships was lower compared to women who did not experience violence.^61^ However, a follow-up study 3 years later showed that in the women who had not been exposed to further violence, this difference in neutralising activity was no longer detectable, whereby cessation of abuse was identified as the key variable for this effect.^62^ This suggests that stress-induced dysregulated immune activity can return to normal under favourable conditions.

### Oral lichen planus

OLP is a chronic inflammatory disease of the oral mucosa. It mainly affects the buccal mucosa, tongue, and gums. Typical lesions are often touch-sensitive or painful and can include bilateral striations, papules, plaques, mucosal atrophies, and blistering.^63^ The underlying pathophysiology of OLP likely involves an antigenic alteration of keratinocytes in the oral mucosa and a subsequent immune response leading to a degeneration of the basal cell layer.^63^^,^^64^ Several studies have found elevated levels of pro-inflammatory cytokines (eg, IL-6, IL-8, IL-18, TNF-α) in the saliva of patients with OLP.^65^ However, the trigger for the expression of planus-specific antigens at the lesion site is still unclear. Mechanical trauma, viral infections, contact allergens, and certain drugs have been discussed as possible causes.^63^^,^^64^ Therefore, the precise aetiology, as well as the extent to which OLP is an autoimmune disease, remains to be clarified.^64^

Previous research has also found evidence of the pathogenic relevance of psychobiological components to the course and prognosis of OLP.^28^^,^^64^^,^^66^ For example, in controlled studies, patients with OLP showed increased levels of psychosocial stress, depression and anxiety,^67^, ^68^, ^69^, ^70^, ^71^ less effective coping,^72^ and more stressful life events.^73^^,^^74^ In addition, patients with OLP have been shown to score high in certain personality traits.^71^ Specifically, patients with OLP are very norm-conscious, conservative, not very emotional, and highly self-controlled (16 Personality Factor Questionnaire [16PF]).^75^ They also have higher levels of depression and tend to somatise, that is, react with physical symptoms in psychologically stressful situations (Minnesota Multiphasic Personality Inventory [MMPI]).^76^ In addition, psychosocial stress has been identified as a common cause of acute relapses in OLP^76^, ^77^, ^78^, ^79^; in a qualitative approach using interviews, patients with OLP reported a subjective worsening of symptoms during times of increased mental stress.^79^

Despite the demonstrated psychosomatic link, the mechanisms mediating psychosocial stress and OLP are poorly understood. One possible but controversial link may again be alterations in the cortisol system of patients. A review of immuno-neuro-endocrine interactions in OLP revealed inconsistent results with regard to HPA axis involvement: 5 out of 9 studies (55.55%) found higher salivary cortisol concentrations in patients with OLP compared to healthy controls; 3 out of 9 studies (33.33%) found no difference; and 1 out of 9 studies (11.11%) found lower cortisol concentrations in patients with OLP.^65^ Similarly, in another study, elevated serum cortisol was only detected in patients with erosive lesions and not in healthy controls or patients with reticular lesions.^76^ Without a doubt, further research is needed to clarify the nature and interplay of the psychobiological components involved in the development and chronification of OLP.

## Methodological considerations

The divergent findings concerning psychological factors and immune activity in oral medicine may be related to fundamental methodological problems in conventional research designs.^4^ Usually, such approaches apply laboratory stress tasks with standardised stressors that do not necessarily match the psychosocial reality of participants. In addition, standardised questionnaires are frequently used to determine psychosocial stressors. Such questionnaires often do not consider the construction and assignment of meaning, subjective aspects that play an essential role in the perception and appraisal of stressful incidents.^80^ Furthermore, conventional nomothetic research designs often focus on cross-sectional relations between variables with a limited temporal scope rather than on temporal relations between consecutive realisations of variables^82^, ^81^ Consequently, such research designs cannot determine the direction of effect between variables (eg, between stressors and symptoms), nor can they yield information on temporal dynamics such as cause-effect delays and complex response patterns.^83^, ^84^, ^85^

**Fig. 2:**
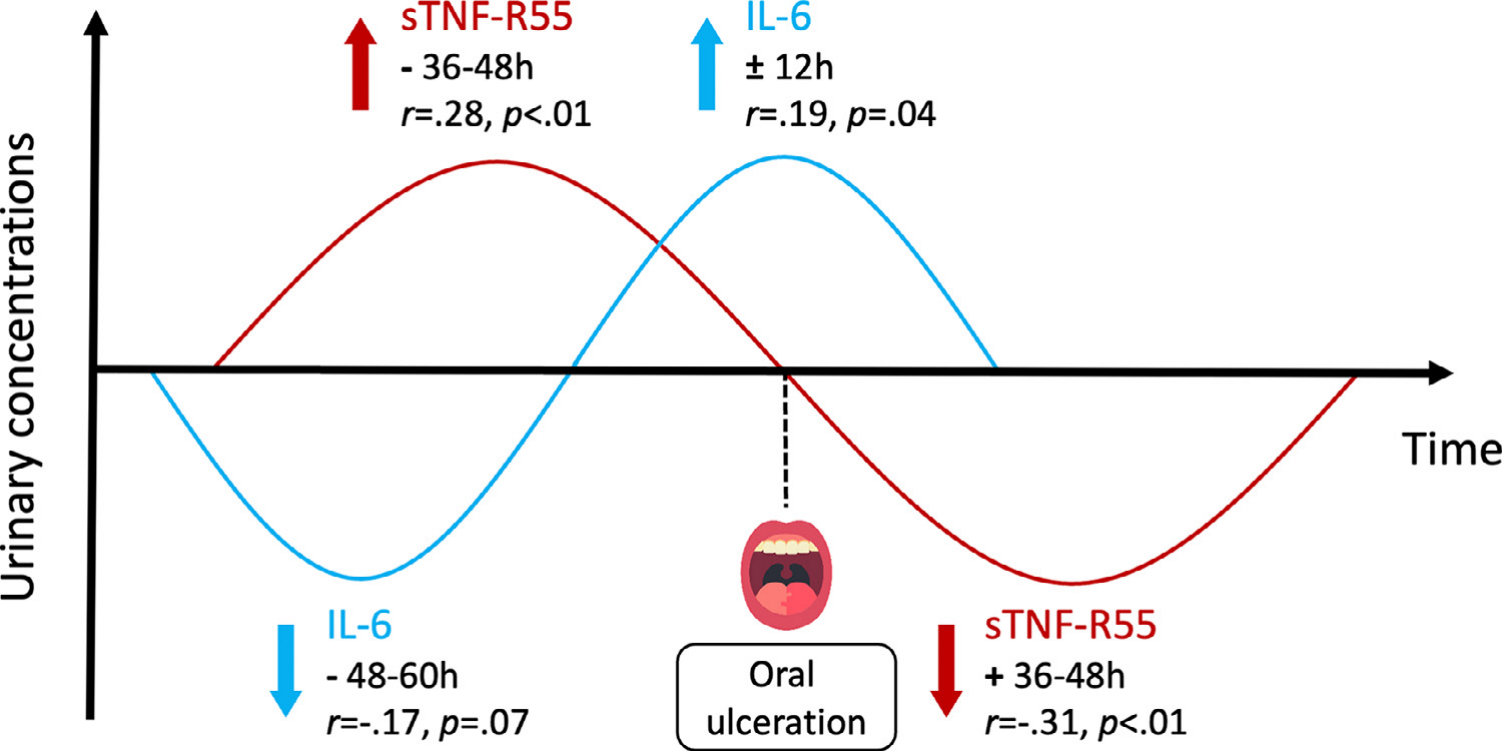
Immune system dynamics before and after oral ulcerations in an integrative single-case study on a patient with systemic lupus erythematosus (SLE). Statistical analysis consisted of autoregressive integrated moving average (ARIMA) modeling and subsequent cross-correlations of symptoms and cytokine time series. For further details, see Schubert et al.^86^, ^87^

We believe that the application of PNI research in oral medicine requires multimodal analysis of single cases based on qualitative (eg, in-depth interviews) and quantitative (eg, time series analysis) data under conditions that are as naturalistic as possible (“life as it is lived”). The integrative single-case design fulfills these criteria.^4^ In one study applying this design, a patient with systemic lupus erythematosus (SLE) collected her entire urine in 12-hour intervals for 56 days (112 twelve-hour intervals), completed questionnaires twice a day, and was interviewed weekly about occurrences during the previous week. Cross-correlational analysis showed feedback mechanisms between oral ulcerations and urinary IL-6 concentrations and between oral ulcerations and soluble tumour necrosis factor receptor type 1 (sTNF-R55) levels (Figure 2). IL-6 concentrations decreased 48 to 60 hours before the occurrence of oral ulcerations and then increased with the appearance of oral ulcerations.^86^ By contrast, sTNF-R55 concentrations increased 36 to 48 hours before the onset of oral ulcerations and decreased 36 to 48 hours after the onset.^87^ In this patient, symptom occurrence was thus associated with both increases and decreases in cytokine levels at different points in time. This kind of cyclic dynamic behaviour in immune system activity may explain the inconsistent or even contradictory results of previous studies on the relationship between symptoms and cytokine levels in SLE.^86^^,^^87^

## Clinical outlook

This review has dealt with the role of PNI in the pathophysiology of periodontitis, herpes labialis, and OLP. Psychosocial stress and associated immuno-neuro-endocrine aberrations were shown to be able to perpetuate and exacerbate disease courses. Thus, the psychosocial reality of patients is clearly relevant and should be part of patient assessment and treatment strategy in oral medicine. Interventions such as stress management programmes, for example, might help prevent oral disease worsening and reduce treatment costs.

To date, however, only limited data are available on the specific benefits of psychosocial interventions in oral medicine. Further well-planned clinical trials are necessary to evaluate the psychosomatic impact of such interventions and their use in routine care.^88^ With regard to periodontitis, herpes labialis, and OLP, research suggests that reducing psychosocial stress and emotional strain may be beneficial.^35^^,^^62^^,^^74^^,^^89^ Promising interventions looked at in recent years have included yoga for periodontal health,^90^ hypnotherapeutic treatment program in herpes labialis,^91^ and psychological counseling in OLP.^92^ In addition, mindfulness-based interventions such as meditation have been recommended to promote overall oral health.^88^^,^^93^

Moreover, as stress can impede wound healing,^45^ it might be beneficial to schedule periodontal surgery or other invasive treatments according to patients' psychosocial circumstances. Performing surgery on a less stressed individual may shorten healing time, lead to fewer complications, decrease the need for medication, and result in shorter hospitalisation.^45^ Patients should also be given information on the impact of stress on oral health and how to minimise psychosocial burden.

## Conclusions

This review has demonstrated that psychosocial influences and immune-neuro-endocrine interactions may be of relevance to oral health. A systems-based approach to oral medicine views humans comprehensively, and PNI provides a suitable empirical framework. The combination of the two may help scientific dentistry move from an dualistic-reductionist view towards a systemic-biopsychosocial paradigm in which the biological condition of the oral cavity may be seen as an expression of a person's life experiences and stress history.

## Conflict of interest

None disclosed.
